# Filamentous Actin in the Nucleus in Triple-Negative Breast Cancer Stem Cells: A Key to Drug-Induced Nucleolar Stress and Stemness Inhibition?

**DOI:** 10.7150/jca.98113

**Published:** 2024-09-03

**Authors:** Xinyu Wang, Runhong Liu, Linli Zhou, Tianyi Liu, Hongyuan Wu, Tiechui Chen, Linya Liu, Xian Zhang, Yiyuan Yang, Yuxuan Guo, Yian Wang, Shujun Fu, Guangchun He, Chanjuan Zheng, Xiyun Deng

**Affiliations:** 1 Key Laboratory of Translational Cancer Stem Cell Research, Department of Pathophysiology, Hunan Normal University School of Medicine, Changsha, Hunan 410013, China.; 2 College of Acupuncture-Moxibustion and Rehabilitation, Hunan University of Chinese Medicine, Changsha 410208, China.

**Keywords:** Filamentous Actin, Nucleus, Triple-Negative Breast Cancer, Nucleolar Stress, Stemness

## Abstract

Actin, primarily a cytoplasmic cytoskeleton protein, is transported in and out of the nucleus with the help of actin-binding proteins (ABPs). Actin exists in two forms, i.e., monomeric globular (G-actin) and polymerized filamentous (F-actin). While G-actin promotes gene transcription by associating with RNA polymerases, F-actin can inhibit this effect in the nucleus. Unexpectedly, we found that lovastatin, an FDA-approved lipid-lowering drug, induces actin redistribution and its translocation into the nucleus in triple-negative breast cancer (TNBC) cancer stem cells. Lovastatin treatment also decreased levels of rRNAs and stemness markers, which are transcription products of RNA Pol I and Pol II, respectively. Bioinformatics analysis showed that actin genes were positively correlated with ABP genes involved in the translocation/polymerization and transcriptional regulation of nuclear actin in breast cancer. Similar correlations were found between actin genes and RNA Pol I genes and stemness-related genes. We propose a model to explain the roles of lovastatin in inducing nucleolar stress and inhibiting stemness in TNBC cancer stem cells. In our model, lovastatin induces translocation/accumulation of F-actin in the nucleus/nucleolus, which, in turn, induces nucleolar stress and stemness inhibition by suppressing the synthesis of rRNAs and decreasing the expression of stemness-related genes. Our model has opened up a new field of research on the roles of nuclear actin in cancer biology, offering potential therapeutic targets for the treatment of TNBC.

## Introduction

The exchange of the components among the various organelles within the cell ensures proper functioning of eukaryotic cells under physiological and pathological conditions. Nuclear translocation of cytoplasmic proteins or other components of the cell has been noted for decades as a means to alter cell functions in response to signaling events or under stressful conditions 1. Actin, one of the most abundant proteins predominantly found in the cytoplasm, has been shown to translocate into the nucleus in many studies 2. While cytoplasmic actin is involved in cell motility, cell division, and cell signaling, nuclear actin is implicated in gene expression regulation, chromatin remodeling, and DNA damage repair under both physiological and pathological conditions 3, 4. The importance of the actin cytoskeleton has been exemplified by the recent finding that the actin cytoskeleton is involved in a novel form of cell death termed disulfidptosis induced by glucose starvation in *SLC7A11^high^* cancer cells 5, 6. A recent report from *Nature* has further demonstrated the importance of nuclear actin in cancer cell biology, showing that nuclear actin mediates resistance to chemotherapy associated with epithelial-to-mesenchymal transition 7.

With regard to gene expression regulation, nuclear actin has been shown to associate with all three types of RNA polymerases (Pol I, II, & III) and regulate gene expression 3. Theoretically, the genes transcribed by RNA Pol I including the precursor form of rRNA (47S/45S) and its mature forms (18S, 28S, and 5.8S) and those transcribed by RNA Pol II are target genes for transcriptional regulation by nuclear actin 8, 9. It should be noted that it is G-actin, rather than F-actin, that is responsible for transcriptional activation in the nucleus 3. Although inconsistent results have been reported 8, the polymerized form of actin (F-actin) has been shown to suppress gene expression by sequestering G-actin in the nucleus 10.

## The mechanisms by which actin enters into and exits from the nucleus

It is known that actin does not possess a classical nuclear localization signal (NLS). Consequently, actin is transported in and out of the nucleus through the nuclear pore complex (NPC) assisted by NLS-containing actin-binding proteins (ABPs). Roughly, 11 ABP genes (*CFL1*/cofilin-1, *CFL2*/cofilin-2, *IPO9*/importin-9, *PFN1*/profilin-1, *XPO6*/exportin-6, *FMN2*/formin-2, *SPIRE1*, *SPIRE2*, *ACTR2*/ARP2, *ACTR3*/ARP3, *EMD*/emerin) are involved in mediating nuclear import/export, assembly, and disassembly of actin 3, 11, 12. Herein, these 11 genes are categorized as “translocation/polymerization (T/P)-related” for the purpose of bioinformatics analysis. Specifically, cofilin (an actin filament-depolymerizing protein) and importin-9 have been found to serve as carriers for nuclear import of actin. Contrarily, profilin (an actin polymerization-promoting protein) and exportin-6 are involved in nuclear export of actin 3. Furthermore, nuclear entry of actin is facilitated by another set of distinct ABPs such as filamins, EMD/emerin, and ARP2/3 12. Five additional ABP genes (*MAL*, *DIAPH1*/mDia1, *DIAPH3*/mDia2, *JMY*, *EP300*/p300) are involved in actin-mediated gene transcriptional regulation 12. In a similar way, these 5 genes are categorized as “transcriptional regulation (TR)-related” for the purpose of bioinformatics analysis in this study.

## 3 Nuclear/nucleolar translocation of F-actin induced by lovastatin: an unexpected discovery

**Figure 1:**
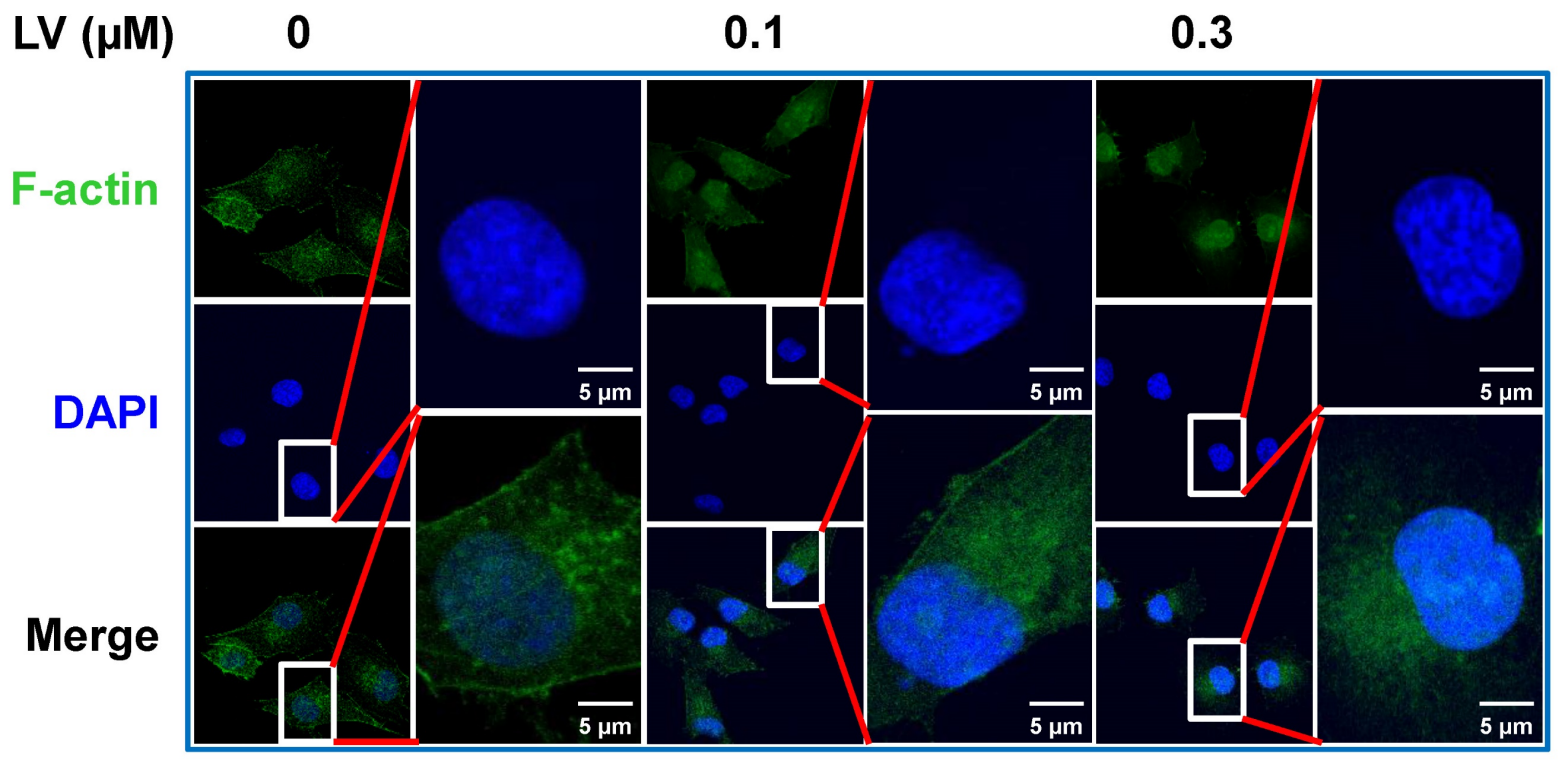
** LV induces translocation of actin into the nucleus.** Representative confocal images of immunofluorescence staining for F-actin with Alexa Fluor488-labeled phalloidin in MDA-MB-231 cancer stem cells after treatment with vehicle or LV (0.1 or 0.3 μM, 48 h). The nucleus was stained blue with DAPI. LV, lovastatin.

Lovastatin (LV) is a naturally derived lipid-lowering drug that has been used in the clinic to treat cardiovascular disease for decades 13. Recently, our group and others have studied the anti-cancer effects of LV in pre-clinical and clinical investigations. In breast cancer, LV preferentially inhibits TNBC over non-TNBC cells through its effects on cancer stem cells 14-16. Interestingly, we found that the cytoskeleton-related pathway is the primary target of LV to exert its stemness-inhibitory effects. Specifically, LV inhibited the formation of pseudopodia and induced the redistribution of F-actin from a diffuse pattern to perinuclear or nuclear localization in TNBC cancer stem cells 14. Moreover, we found that LV could induce nucleolar stress and inhibit stemness in TNBC cancer stem cells in our recent study 16 and as described below. In this short report, we further analyzed the distribution of F-actin in LV-treated MDA-MB-231 TNBC cancer stem cells and found the accumulation of phalloidin-stained F-actin in the nucleus and, in particular, the nucleolus upon LV treatment (**Figure 1**).

**Figure 2:**
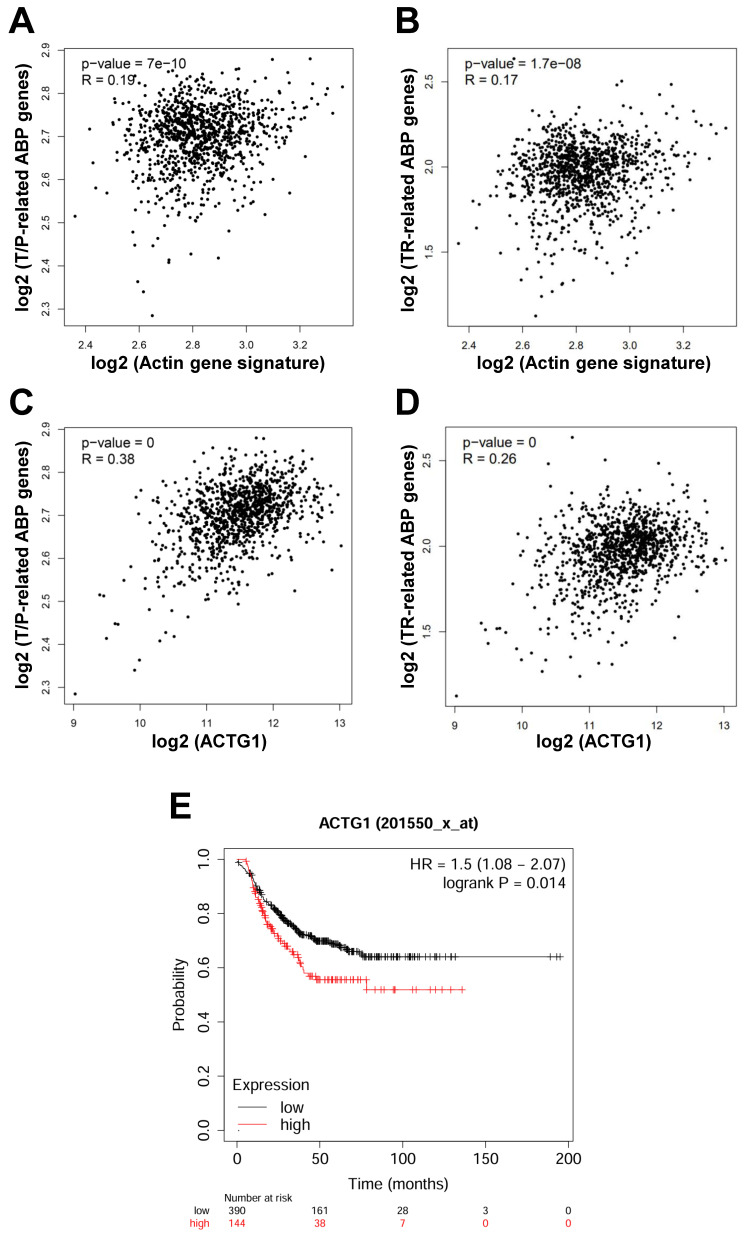
** Actin is positively correlated with ABP genes in breast cancer and high expression of *ACTG1* predicts poor prognosis in TNBC patients.** (**A** and **B**) Correlation between the signature consisting of 6 actin genes and the 11 T/P-related ABP genes (**A**) and the 5 TR-related ABP genes (**B**) associated with nuclear actin in breast cancer analyzed through GEPIA2 (http://gepia2.cancer-pku.cn/#index). (**C** and **D**) Correlation between the *ACTG1* gene and the 11 T/P-related ABP genes (**C**) and the 5 TR-related ABP genes (**D**) in breast cancer analyzed through GEPIA2. (**E**) Kaplan-Meier survival analysis of TNBC patients for *ACTG1* between the high- and low-expression groups (https://kmplot.com/analysis/index.php?p=service&cancer=breast). ABPs, actin-binding proteins; T/P, translocation/polymerization; TR, transcriptional regulation.

As mentioned above, actin is transported in and out of the nucleus in the form of G-actin. In the nucleus, G-actin is polymerized into F-actin to perform gene expression-regulating functions 17. Therefore, we speculate that LV promotes disassembly of the F-actin into a globular-like structure around the nucleus, translocation of actin into the nucleus, and reassembly back into the filamentous structure in the nucleus. In this process, ABPs play important roles through binding, assembly/disassembly, transport of actin between the cytoplasm and the nucleus. We thus performed correlation analysis between the 6 actin genes (*ACTA1*, *ACTA2*, *ACTB*, *ACTC1*, *ACTG1*, and *ACTG2*) 18 and the ABPs involved in translocation/polymerization and transcriptional regulation of nuclear actin in breast cancer through the GEPIA2 web-based platform. We found that the signature consisting of the 6 actin genes was positively correlated with the signature consisting of the 11 ABPs mediating actin translocation/polymerization (R = 0.19, *P* = 7×10^-10^) (**Figure 2A**) and the signature consisting of 5 ABPs facilitating transcriptional regulation of actin in the nucleus (R = 0.17, *P* = 1.7×10^-8^) (**Figure 2B**).

Because 2 (*ACTB*, *ACTG1*) out of the 6 actin genes are expressed in non-muscle cells 19, we further analyzed the correlation of these 2 actin genes with the above ABP genes in breast cancer. While the result of *ACTB* was not convincing, the expression of *ACTG1* was consistently positively correlated with that of the ABP genes involved in actin translocation/polymerization (R = 0.38, *P* = 0) (**Figure 2C**) and the ABP genes involved in regulating transcriptional regulation of nuclear actin (R = 0.26, *P* = 0) (**Figure 2D**). To reveal the importance of *ACTG1* in TNBC patients, we conducted Kaplan-Meier survival analysis through publically available databases. We found that high expression of *ACTG1* (and 5 other actin genes as well) was associated with poor prognosis in TNBC patients (**Figure 2E**). These results suggest that dysregulation of the actin cytoskeleton pathway plays an important role in TNBC pathogenesis and prognosis, which might be a critical target for drugs like LV.

## Functions of nuclear/nucleolar actin induced by lovastatin in TNBC cancer stem cells

Over half a century ago, Jones *et al.* described a loose structure of “uniform threads” within the nucleus of frog embryos after exposure to actinomycin D 20, 21. Actinomycin D is an antibiotic known to bind DNA and inhibit DNA-dependent RNA transcription in the nucleolus, which is the main site of ribosome biogenesis 22. Later, F-actin was found be translocated into the nucleus in places where DAPI staining is weak, which is presumably the nucleolus, upon cellular stress, such as treatment with doxorubicin 23, 24. These findings link drug-induced F-actin translocation to nucleolar stress, dysfunction of the ribosome biogenesis process 22.

Our recent study has demonstrated that LV induced nucleolar stress and caused stemness inhibition in TNBC cancer stem cells 16. Here, we confirmed nucleolar stress induced by LV using qRT-PCR analysis, showing that the levels of 28S and 5.8S rRNAs, the gene products transcribed from RNA Pol I, were suppressed by LV in MDA-MB-231 cancer stem cells (**data not shown**). Because nuclear actin associates with all three types of RNA Pols, we reckon nuclear F-actin also mediates stemness-inhibitory functions of LV in TNBC cancer stem cells through its action on RNA Pol II. Upon re-verification, we discovered that LV treatment indeed reduced the protein levels of stemness markers in MDA-MB-231 cancer stem cells, including β-catenin and Nanog, the gene products transcribed from RNA Pol II (**data not shown**).

**Figure 3:**
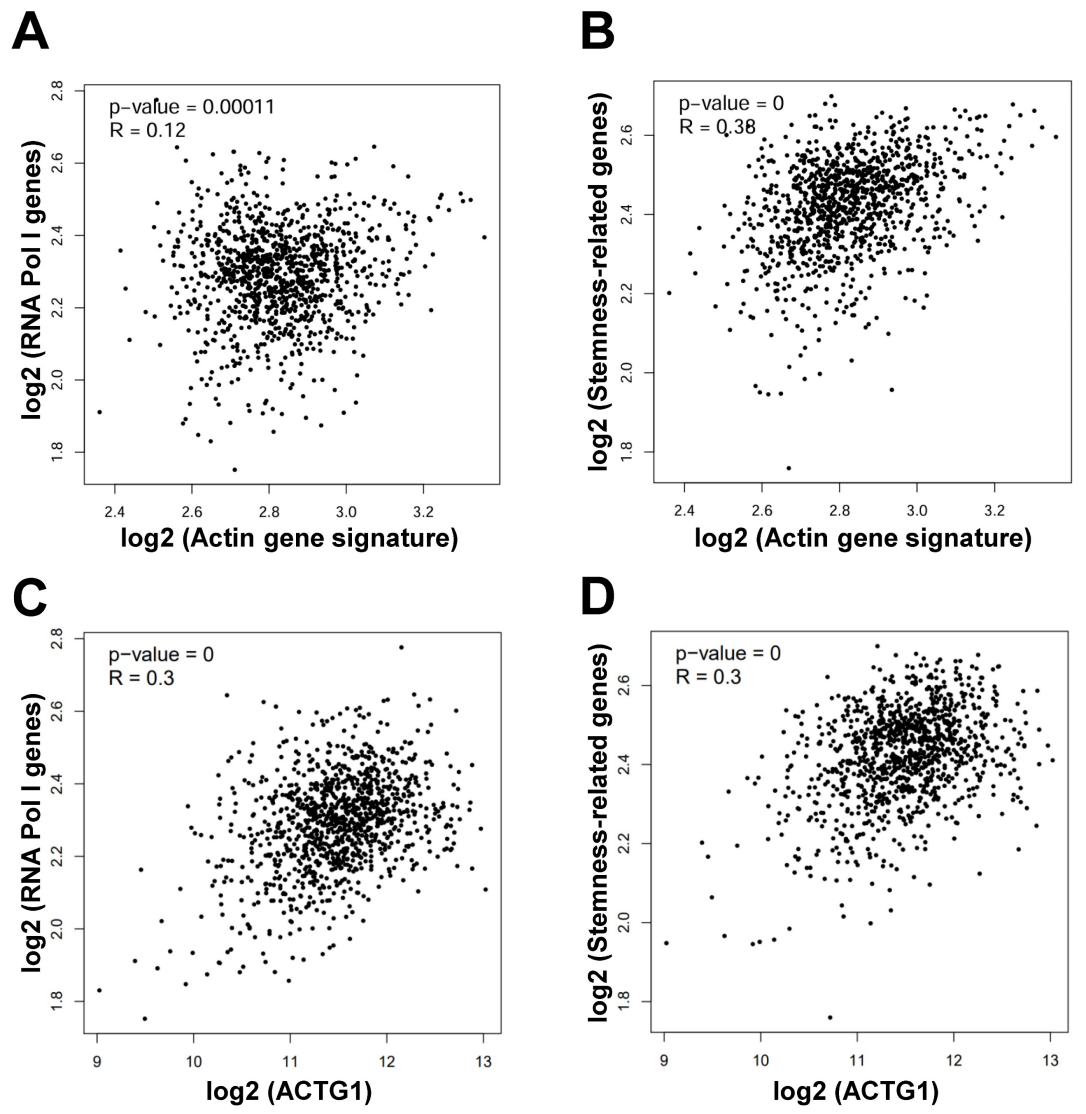
** Actin genes are positively correlated with the RNA Pol I genes and stemness-related genes in breast cancer.** (**A** and **B**) Correlation between the actin gene signature and the RNA Pol I genes (**A**) and stemness-related genes (**B**) in breast cancer analyzed through GEPIA2. (**C** and **D**) Correlation between the *ACTG1* gene and the RNA Pol I genes (**C**) and stemness-related genes (**D**) in breast cancer analyzed through GEPIA2.

In order to reveal the correlation of actin genes with genes involved in ribosome biogenesis, we performed correlation analysis through GEPIA2 in breast cancer. We found that the actin gene signature was positively correlated with the signature consisting of the 4 RNA Pol I genes (*POLR1A*, *POLR1B*, *POLR1C*, *POLR1E*) involved in ribosome biogenesis 16 (R = 0.12, *P* = 0.00011) (**Figure 3A**). Likewise, the actin gene signature was positively correlated with the signature consisting of the 15 stemness-related genes (*MYC*, *ALDH18A1*, *ALDH1A1*, *SMAD2*, *SMAD3*, *CD44*, *PROM1*, *EpCAM*, *ITGB1*, *NANOG*, *SOX2*, *TCF7L2*, *POU5F1*, *KLF4*, *SDC1*) 25 (R = 0.38, *P* = 0) (**Figure 3B**). Similarly, the *ACTG1* gene was positively correlated with the 4 RNA Pol I genes (R = 0.3, *P* = 0) (**Figure 3C**) and the 15 stemness-related genes (R = 0.3, *P* = 0) (**Figure 3D**). These results suggest that actin particularly *ACTG1* presumably mediates nucleolar stress and stemness inhibition induced by LV in TNBC cancer stem cells.

## Our proposed model

**Figure 4:**
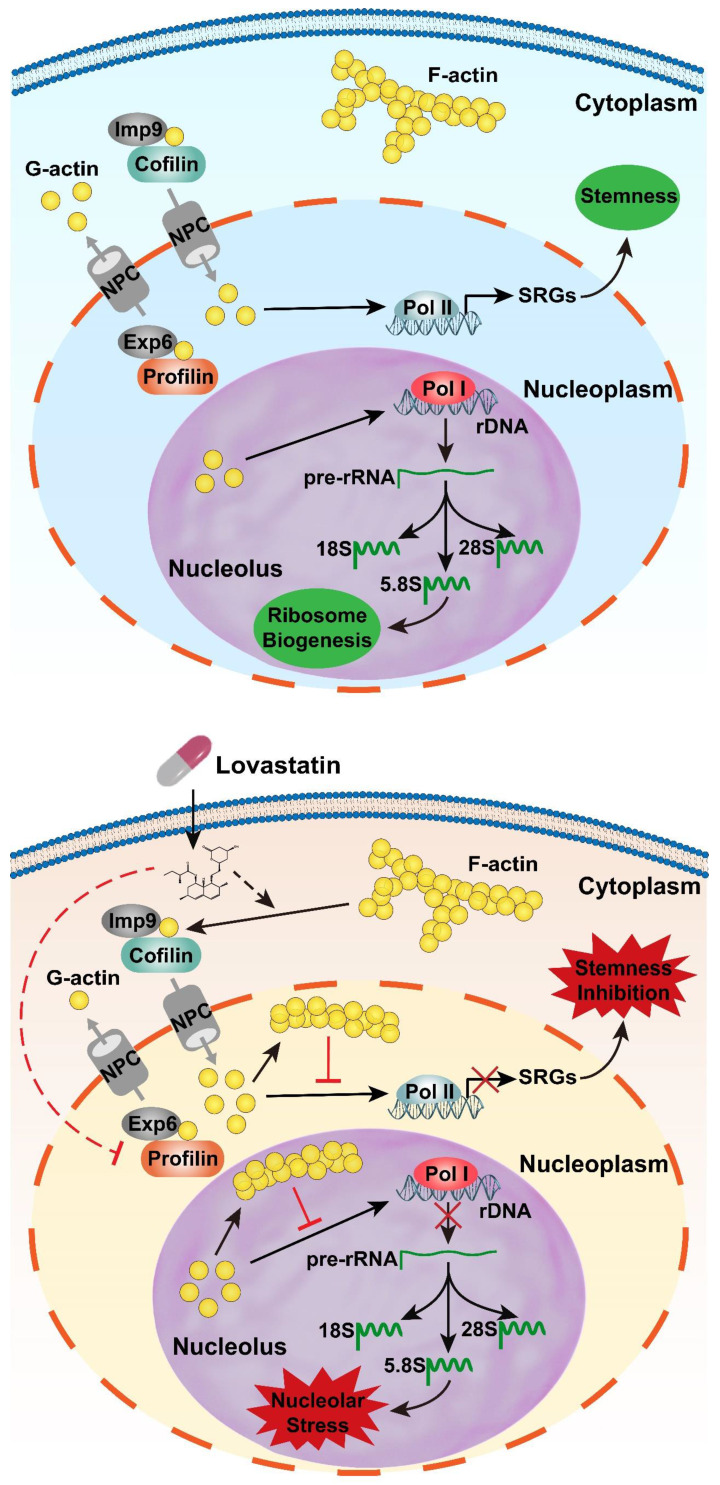
** A model depicting LV-induced nuclear translocation of actin followed by nucleolar stress and stemness inhibition. Top**: In the absence of LV, actin primarily exists as polymers (F-actin) in the cytoplasm and as monomers (G-actin) within the nucleus. G-actin in the nucleus promotes gene transcription through its association with all three types of RNA polymerases. The genes transcribed from RNA Pol I are precursor rRNA and its mature forms (18S, 5.8S, 28S). The genes transcribed from RNA Pol II include the stemness-related genes, among others. As a result, the cells display “normal” ribosome biogenesis and stemness properties. **Bottom**: In the presence of LV, F-actin is depolymerized into G-actin by cofilin, which mediates G-actin nuclear entry together with Imp9. In the nucleus, G-actin molecules aggregate into F-actin, which inhibits G-actin-mediated gene transcription. Ultimately, nuclear F-actin promotes nucleolar stress through suppression of RNA Pol I-mediated transcription of rRNAs and causes stemness inhibition through suppression of RNA Pol II-mediated transcription of stemness-related genes. Solid lines indicate known pathways; dotted lines denote hypothetical effects of LV proposed in this research communication. ABPs, actin-binding proteins; Exp6, exportin 6; Imp9, importin 9; LV, lovastatin; NPC, nuclear pore complex; RNA Pol I, RNA polymerase I; RNA Pol II, RNA polymerase II; SRGs, stemness-related genes.

Based on the results from our previous publications and the findings from this short report together with the literature reports, we propose a model to elucidate the roles of LV in nucleolar stress induction and stemness inhibition in TNBC cancer stem cells. In this model, LV promotes translocation/accumulation of actin into the nucleus through the NPC assisted by cofilin/importin-9 for enhanced nuclear entry and profilin/exportin-6 for decreased nuclear exit. Upon entering the nucleus, the transported G-actin is polymerized back into F-actin. On the one hand, nuclear F-actin promotes nucleolar stress through suppression of RNA Pol I-mediated transcription of rRNAs; on the other hand, nuclear F-actin promotes stemness inhibition through suppression of RNA Pol II-mediated transcription of stemness-related genes (**Figure 4**). Collectively, nuclear translocation of actin plays a key role in inhibiting the malignant phenotype in TNBC cancer stem cells. Understanding the molecular details behind each step of the process of LV-induced nuclear translocation of actin will present a clear picture on how LV causes suppression of TNBC cancer stem cells and provide a rationale to design more robust TNBC cancer stem cell-targeting therapeutic strategies.
